# Urethral reconstruction with autologous urine-derived stem cells seeded in three-dimensional porous small intestinal submucosa in a rabbit model

**DOI:** 10.1186/s13287-017-0500-y

**Published:** 2017-03-09

**Authors:** Yang Liu, Wenjun Ma, Bo Liu, Yangcai Wang, Jiaqiang Chu, Geng Xiong, Lianju Shen, Chunlan Long, Tao Lin, Dawei He, Denis Butnaru, Lyundup Alexey, Yuanyuan Zhang, Deying Zhang, Guanghui Wei

**Affiliations:** 1 Department of Urology, Children’s Hospital of Chongqing Medical University, Chongqing, 400014 China; 2 Ministry of Education Key Laboratory of Child Development and Disorders, Chongqing Key Laboratory of Pediatrics, China International Science and Technology Cooperation base of Child development and Critical Disorders, Chongqing Key Laboratory of Child Urogenital Development and Tissue Engineering, Chongqing, 400014 China; 3 Chongqing Engineering Research Center of Stem Cell Therapy, Children’s Hospital of Chongqing Medical University, Chongqing, China; 4 Research Institute for Uronephrology, Sechenov First Moscow State Medical University, Moscow, 119991 Russia; 5 Biomedical Research Department of Institute of Molecular Medicine, Sechenov First Moscow State Medical University, Moscow, 119991 Russia; 6 Wake Forest Institute for Regenerative Medicine, Wake Forest School of Medicine, Winston-Salem, NC 27101 USA

**Keywords:** Urine-derived stem cells, Tissue engineering, Urethral reconstruction

## Abstract

**Background:**

Urethral reconstruction is one of the great surgical challenges for urologists. A cell-based tissue-engineered urethra may be an alternative for patients who have complicated long strictures and need urethral reconstruction. Here, we demonstrated the feasibility of using autologous urine-derived stem cells (USCs) seeded on small intestinal submucosa (SIS) to repair a urethral defect in a rabbit model.

**Methods:**

Autologous USCs were obtained and characterized, and their capacity to differentiate into urothelial cells (UCs) and smooth muscle cells (SMCs) was tested. Then, USCs were labeled with PKH67, seeded on SIS, and transplanted to repair a urethral defect. The urethral defect model was surgically established in New Zealand white male rabbits. A ventral urethral gap was created, and the urethral mucosa was completely removed, with a mean rabbit penile urethra length of 2 cm. The urethral mucosal defect was repaired with a SIS scaffold (control group: SIS with no USCs; experimental group: autologous USC-seeded SIS; *n* = 12 for each group). A series of tests, including a retrograde urethrogram, histological analysis, and immunofluorescence, was undertaken 2, 3, 4, and 12 weeks after the operation to evaluate the effect of the autologous USCs on urethral reconstruction.

**Results:**

Autologous USCs could be easily collected and induced to differentiate into UCs and SMCs. In addition, the urethral caliber, speed of urothelial regeneration, content of smooth muscle, and vessel density were significantly improved in the group with autologous USC-seeded SIS. Moreover, inflammatory cell infiltration and fibrosis were found in the control group with only SIS, but not in the experimental autologous USC-seeded SIS group. Furthermore, immunofluorescence staining demonstrated that the transplanted USCs differentiated into UCs and SMCs in vivo.

**Conclusions:**

Autologous USCs can be used as an alternative cell source for cell-based tissue engineering for urethral reconstruction.

## Background

Reconstruction of the adult and pediatric male penile urethra has been one of the great surgical challenges for urologists, especially in cases of recurrent hypospadias and long urethral defects after trauma. More than 200 different reconstructive methods for urethral stricture disease or congenital absence have been described, with a variety of donor tissues, such as free skin grafts [1], bladder mucosa [2], buccal mucosa [3], and lingual mucosa [4], proving successful for urethral reconstruction. However, in many cases, an adequate amount of donor tissue is not available [5]. In addition, substantial donor site morbidity, including submucosal scarring, pain, numbness, injury to salivary ducts, and limitation of mandibular movement, has been reported [6]. Furthermore, complications, such as fistulae, hair growth, graft shrinkage, strictures, stone formation, and diverticulum, occur in the long term following the procedure [7].

The use of tissue-engineered grafts to repair urethral defects is considered to be an approach with therapeutic potential. Natural collagen materials, such as bladder submucosa (BSM) [8, 9], small intestinal submucosa (SIS) [10], or collagen type I matrix [11], have already been successfully used in urological tissue engineering for short urethral defects both experimentally [12] and clinically [13]. Previous studies [8, 14] demonstrated that a SIS decellularization procedure including a 5% peracetic acid (PAA) treatment step leads to higher porosity and larger pore size. These porous microstructures allow cell infiltration into the matrix and have fewer heterogeneous cellular proteins, thereby preventing complications, such as inflammatory reactions, fibrosis, calcification, and graft shrinkage.

Another critical element required for successful urethral tissue engineering is the cell source. A subpopulation of stem cells can be easily isolated from human voided urine [15–17], i.e., urine-derived stem cells (USCs). USCs are multipotent cells that have been widely demonstrated to terminally differentiate into a variety of different cell types, including smooth muscle cells (SMCs) and uroepithelial, endothelial, osteogenic, adipogenic, chondrogenic, and neural lineages [18]. Furthermore, autologous USCs can be harvested using a simple, safe, low-cost, and non-invasive procedure. Thus, USCs may be an excellent alternative cell source for urological tissue engineering applications.

Therefore, we cultured autologous rabbit USCs before surgery and seeded them onto a 5% PAA-treated SIS scaffold to reconstruct the urethra in rabbits. This cell-based tissue-engineered urethra may be useful to patients with complicated long strictures who need urethral reconstruction.

## Methods

### SIS scaffold preparation

Fresh porcine small intestines were obtained and chemically modified according to a protocol published previously by Liu et al. [9]. The mucosa and serosa were mechanically removed and washed in distilled water. For decellularization, SIS samples were treated with 5% PAA for 4 h with agitation on an orbital shaker at 200 rpm at 4 °C. After decellularization, they were treated with 1% Triton X-100 for a further 2 days. Then, the SIS was washed in distilled water for another 72 h. Finally, the SIS scaffold was sterilized using 0.1% PAA prepared in 20% ethanol for 2 h, rinsed six times with sterile phosphate buffer solution for 20 min each time, and stored in sterile phosphate buffer solution containing 100 U/ml penicillin and 0.1 mg/ml streptomycin at 4 °C until needed.

### Scanning electron microscopy

Fresh SIS and 5% PAA-treated SIS were fixed in 2.5% glutaraldehyde. The SIS samples were frozen at –80 °C for 24 h, sputter-coated with gold, and mounted for analysis. Electron micrographs of mucosa slides were obtained with an acceleration voltage of 25.0 kV at a magnification of 400× using a Hitachi S-3000 N Scanning Electron Microscope (Hitachi Technologies America, Pleasanton, CA, USA).

### DNA content

For measurement of residual DNA, fresh SIS and 5% PAA-treated SIS samples were dabbed dry with tissue paper and weighed. Samples were minced, and any remaining nucleic acids were extracted using a commercially available kit (DNeasy™, Qiagen, Valencia, CA, USA). The DNA concentration in each sample was calculated using a UV spectrophotometer (ND-2000, NanoDrop Technologies, Wilmington, DE, USA) to measure the absorbance at 260 nm. The DNA yield was normalized to the initial wet weight of the samples.

### Isolation of autologous rabbit USCs

Urine and bladder irrigation solution samples (average total volume of approximately 60 ml/sample) were collected from every rabbit that was to undergo surgery. Each sample was centrifuged at 500 g for 5 min to collect the cells, and the supernatant was carefully discarded. The pelleted cells were gently resuspended in medium (a 1:1 mixture of KSFM and embryonic fibroblast medium (EFM)) and plated in 24-well plates for single-cell cultures. The medium was changed every 2 days after the initial 72 h, and cells were passaged at 70–80% confluency [15].

### Urothelial and smooth muscle differentiation of USCs

For urothelial differentiation, USCs were plated at a density of 3000 cells/cm^2^ in medium composed of equal amounts of KSFM and EFM containing 2% serum and 30 ng/ml epidermal growth factor (R&D Systems, Minneapolis, MN, USA) and cultured for 14 days [19]. The medium was replaced every 3 days, and, on day 14, the cells were analyzed by immunofluorescence (IF) staining and Western blotting for urothelial cell (UC)-specific markers (AE1/AE3).

For SMC differentiation, USCs were plated at a density of 2000 cells/cm^2^ in myogenic differentiation medium composed of equal amounts of DMEM and EFM containing 10% fetal bovine serum (FBS) as well as 2.5 ng/ml transforming growth factor (TGF)-1 and 5 ng/ml platelet-derived growth factor (PDGF)-BB (R&D systems) [18]. The medium was replaced every 3 days, and, on day 14, the cells were analyzed by IF staining and Western blotting for the SMC-specific marker myosin.

### Autologous USC-seeded SIS scaffold preparation and proliferation assay

A suspension of USCs was seeded on the serosal side of the SIS at a density of 2 × 10^5^ cells/cm^2^. On the third day, the scaffolds were inverted and a similar number of USCs was seeded onto the mucosal side of the SIS. Every 3 days, the scaffolds were inverted. After 14 days of culture, the USC-seeded, 5% PAA-treated SIS scaffolds were ready to use for the experiment.

To analyze the cytotoxicity of the 5% PAA-treated SIS scaffold, CCK-8 assays were performed. USCs cultured in 96-well plates with 5% PAA-treated SIS were compared with USCs cultured in 96-well plates without 5% PAA-treated SIS. Briefly, 1000 USCs were seeded in each well of 96-well plates. After the cells adhered, the medium was removed, and medium containing 5% PAA-treated SIS was added. Cells cultured without scaffolds were used as the control.

### Cell labeling and tracking

To detect differentiation of the seeded USCs, USCs were labeled with a PKH67 green fluorescent labeling kit (Sigma Aldrich) following the manufacturer’s protocol. Briefly, USCs were incubated with PKH67 for 5 min and washed four times to remove excess dye. After being labeled, the cells were seeded into SIS scaffolds and transplanted into rabbit urethras. IF was performed 2, 3, and 4 weeks after transplantation for markers of the urothelium (AE1/AE3; Abcam) and smooth muscle (myosin; Abcam).

### Rabbit urethra reconstruction utilizing the SIS scaffold

All pre- and post-animal procedures were performed in accordance with guidelines set forth by the Ethics Committee of Chongqing Medical University, and were approved by this committee (license number: SCXK[YU]20110016). Twenty-four New Zealand white male rabbits (2.0–2.5 kg) were randomly divided into two groups, each containing 12 animals. Autologous USCs had been obtained from each of these rabbits and cultured. The experimental group received urethroplasty with an autologous USC-seeded 5% PAA-treated SIS scaffold, and the control group received urethroplasty with a 5% PAA-treated SIS scaffold alone. Animals were anesthetized with pentobarbital (30 mg/kg) administered intravenously and lidocaine (6 mg/kg) administered subcutaneously to decrease any pain during and after the surgery. A ventral urethral gap was created a mean of 2 cm from the external urethral orifice, and the urethral mucosa was exposed and completely removed, with a mean penile urethra length of 2 cm. The urethral mucosal defect was repaired with a SIS scaffold (control group: 5% PAA-treated SIS alone with no USCs; experimental group: autologous USC-seeded 5% PAA-treated SIS; *n* = 12 for each group). The SIS scaffold ends were anastomosed with the native ends of the urethral tissue using 6-0 absorbable sutures in a water-tight fashion. The wound was closed in layers in a routine fashion. An 8 F silicone catheter was indwelled and fastened with 6-0 absorbable sutures to provide bladder drainage for the 14 days following the operation. Each group was sacrificed at the 2, 3, 4, or 12 week time point. At 12 weeks, the animals in the two groups were assessed with retrograde urethrograms.

### Histological analysis

Fresh SIS, 5% PAA-treated SIS, USC-seeded 5% PAA-treated SIS, and urethral specimens were fixed in 10% buffered formalin, embedded in paraffin, and processed for histology (hematoxylin and eosin (H&E) staining, 4′,6-diamidino-2-phenylindole (DAPI) staining, Masson’s trichrome staining, immunohistochemistry (IHC), and IF). IHC was performed using monoclonal antibodies against the urothelium (AE1/AE3; Abcam), smooth muscle (myosin; Abcam), and vascular endothelium (CD31; Abcam). Briefly, consecutive 4.0-μm sections were prepared using a cryostat and incubated overnight at 4 °C with the following primary antibodies: anti-AE1/AE3 diluted 1:75, anti-myosin diluted 1:100, and anti-CD31 diluted 1:150. Then, sections were washed with phosphate-buffered saline (PBS) and incubated with the appropriate secondary antibody diluted 1:200 for 60 min at room temperature. Finally, the slides were rinsed twice with PBS and counterstained with hematoxylin or DAPI. IF was performed using monoclonal antibodies against the urothelium (AE1/AE3; Abcam) and smooth muscle (myosin; Abcam). Briefly, frozen sections (6.0 μm) or cells were fixed in 4% paraformaldehyde and blocked with 5% bovine serum albumin. Subsequently, the sections were treated overnight at 4 °C with the following primary antibodies: anti-AE1/AE3 diluted 1:75 or anti-myosin diluted 1:100. They were then incubated with a secondary antibody (IgG labeled with fluorescein) and counterstained with DAPI.

### Statistical analysis

The data are presented as mean and standard error of the mean for each group. Individual comparisons were made between groups using two-tailed Student’s *t* tests. Differences were considered statistically significant for *P* values < 0.05.

## Results

### Histological analysis of the SIS

**Fig. 1:**
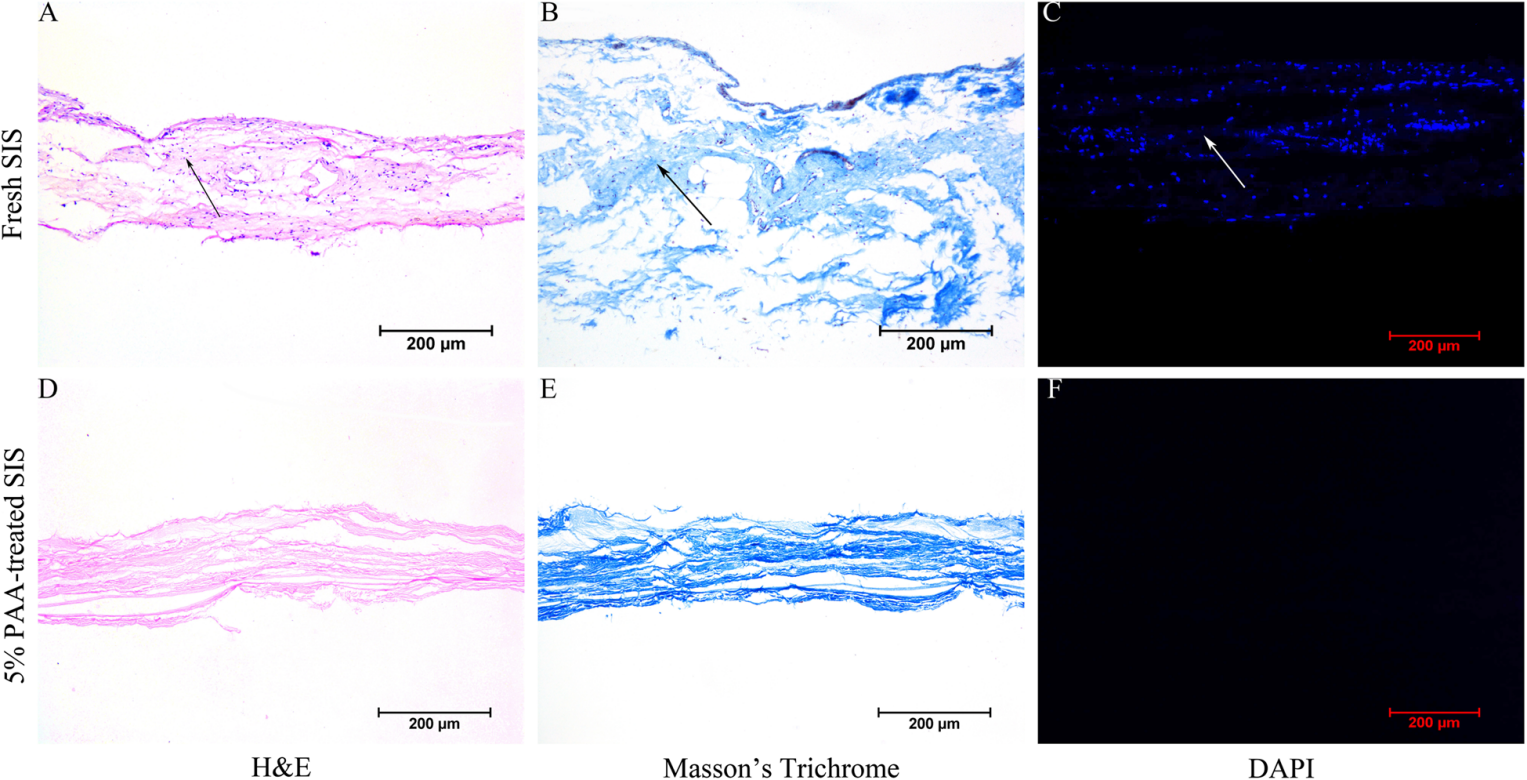
Histological analysis of the SIS. H&E (**a** and **d**), Masson’s trichrome (**b** and **e**), and DAPI staining (**c** and **f**) of cross-sections of fresh SIS (**a**, **b**, and **c**) and 5% PAA-treated SIS (**d**, **e**, and **f**). Nuclear material was found in fresh SIS tissue (*arrows*; **a**, **b**, and **c**). However, treatment with 5% PAA completely removed the nuclear material (**d**, **e**, and **f**). *Scale bar* = 200 μm. *DAPI* 4′,6-diamidino-2-phenylindole, *H&E* hematoxylin and eosin, *PAA* peracetic acid, *SIS* small intestinal submucosa

Examination of fresh SIS stained with H&E, Masson’s trichrome, and DAPI (Fig. 1) showed that, without 5% PAA, some nuclear material remained in the SIS. By contrast, after treatment with 5% PAA, the nuclear material was almost completely removed and the tissue structure became visibly more porous.

### Scanning electron microscopy

**Fig. 2:**
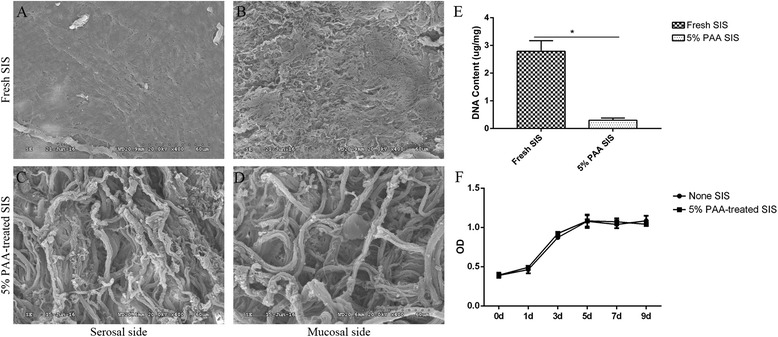
Scanning electron microscopy, DNA content analysis, and proliferation assay. Electron micrographs of the mucosal (**b** and **d**) and serosal (**a** and **c**) sides of fresh SIS (**a** and **b**) and 5% PAA-treated SIS (**c** and **d**). A high level of porosity and large pore size were evident after treatment with 5% PAA. *Scale bar* = 60 μm. The DNA content analysis (**e**) showed a decrease in DNA content after treatment with 5% PAA. A significant difference in the DNA content was observed between the two groups (*P < 0.05). **f** Analysis of cell growth. The OD values in the scaffold groups were similar to those in the control group (*P* > 0.05). *d* days, *OD optical density*, *PAA* peracetic acid, *SIS* small intestinal submucosa

Scanning electron micrographs (Fig. 2a, b, c, and d) showed dense ultrastructure on the mucosal and serosal sides of fresh SIS. By contrast, after treatment with 5% PAA, porosity was visible on the surface. The diameter of these pores ranged from 50 to 150 nm, greater than the average cell diameter, which allowed homogenous cell infiltration into the three dimensional (3D) scaffold [9].

### DNA content and proliferation assay

The DNA content analysis (Fig. 2e) showed a decrease in DNA content after 5% PAA treatment. The difference in DNA content between the two groups was significant (*P* < 0.05). The USCs grew well in medium containing the 5% PAA-treated SIS scaffold, and the OD (optical density) values in the scaffold groups were similar to those in the control group (*P* > 0.05) (Fig. 2f).

### Characteristics and multipotent differentiation of USCs

**Fig. 3:**
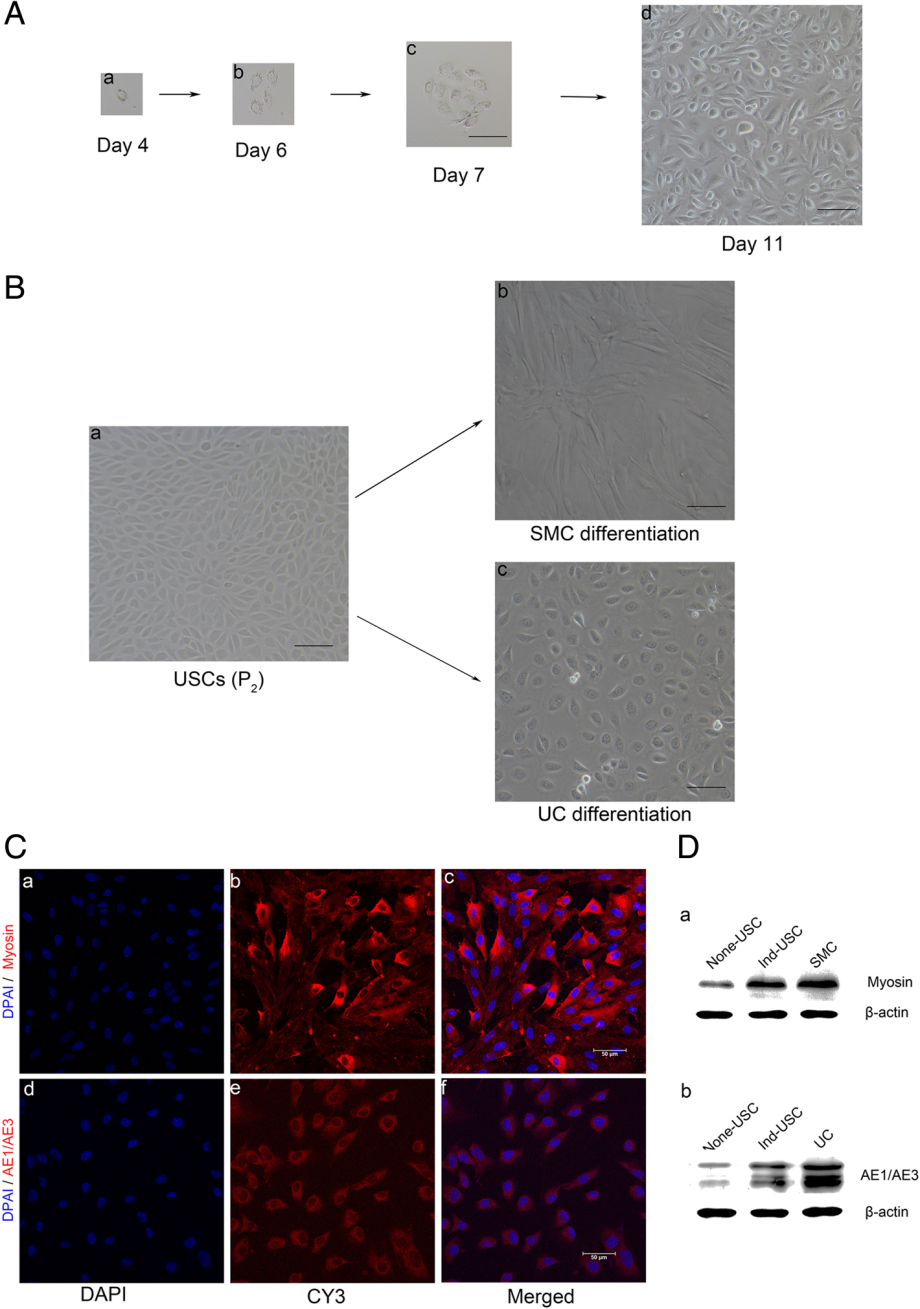
Characteristics and multipotent differentiation of USCs. **a** Single USCs can be expanded to a large population. Single cells appeared on day 4 (**a** *a*), clone formation was observed on day 6 and 7 (**a** *bc*), and cell confluency was achieved on day 11 (**a** *d*). *Scale bar* = 100 μm. **b** USCs (passage 2) were induced to differentiate into two distinct lineages. The morphology of USCs (**b** *a*) is shown after induction with SMC induction media (**b** *c*) and UC induction media (**b** *b*) for 14 days. *Scale bar* = 100 μm. **c** IF staining of myogenic-induced (**c** *a*–**c** *c*) and urothelial-induced (**c** *d*–**c** *f*) USCs. Specific marker staining (*red*) and nuclear staining by DAPI (*blue*) are shown. *Scale bar* = 50 μm. **d** Detection of the SMC-specific marker (myosin; **d** *a*) and UC-specific markers (AE1/AE3; **d** *b*) by Western blotting. Specific bands are seen in lanes with proteins from induced USCs and the positive control. *DAPI* 4′,6-diamidino-2-phenylindole, *SMC* smooth muscle cell, *UC* urothelial cell, *USC* urine-derived stem cell

Single USCs were observed 3–4 days after initial seeding (Fig. 3Aa). These cells then formed clones within the next 4–7 days (Fig. 3Ab, c), and single USCs were able to expand into a large population (Fig. 3Ad). They exhibited a characteristic “rice grain”-like morphology (Fig. 3Ad). After several passages, the cells retained their “rice grain” morphology. USCs that differentiated into smooth muscle showed a spindle-like morphology (Fig. 3Bb) and expressed the skeletal muscle-specific protein myosin (Fig. 3Cc).Cells that differentiated into urothelium exhibited a cobblestone-like morphology (Fig. 3Bc) and expressed the urothelium-specific proteins AE1/AE3 (Fig. 3Cf). Western blot analysis revealed that myosin and AE1/AE3 (Fig. 3D) were present in induced urine-derived cells.

### Seeding of autologous USCs onto SIS scaffolds

**Fig. 4:**
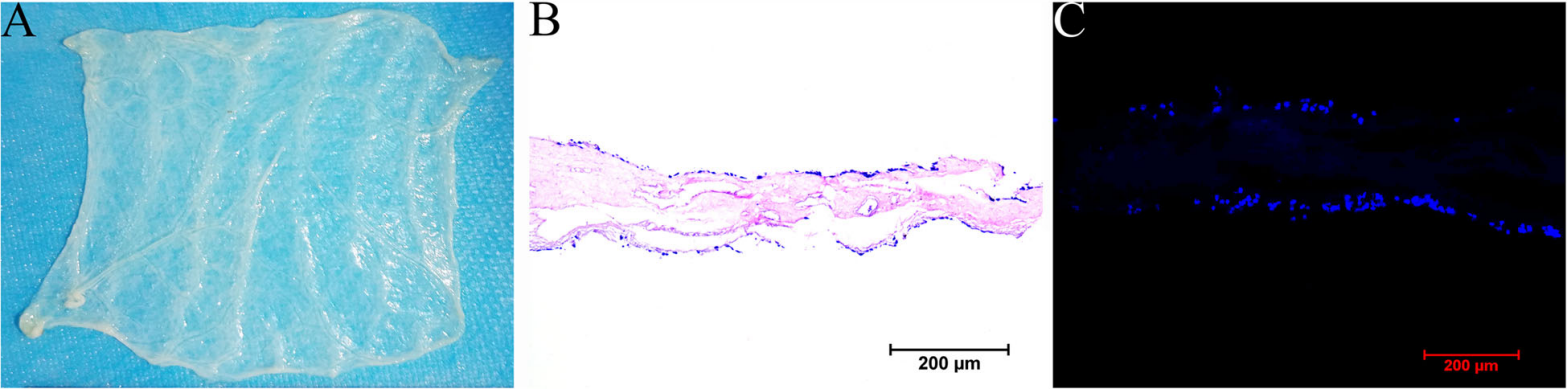
Seeding of SIS scaffolds with autologous USCs. **a** Collagen-based scaffolds derived from porcine SIS were mechanically and chemically treated to remove cells. H&E (**b**) and DAPI (**c**) staining showed that there were two to four layers of USCs attached to both sides of the 5% PAA-treated SIS scaffold after seeding. *Scale bar* = 200 μm

Macroscopy showed that collagen-based scaffolds derived from porcine SIS had been mechanical and chemically treated to remove cells (Fig. 4a). Examination of the H&E and DAPI (Fig. 4b and c) staining showed that there were two to four layers of USCs attached to both sides of the 5% PAA-treated SIS scaffold after seeding.

### Macroscopy and retrograde urethrogram evaluation

**Fig. 5:**
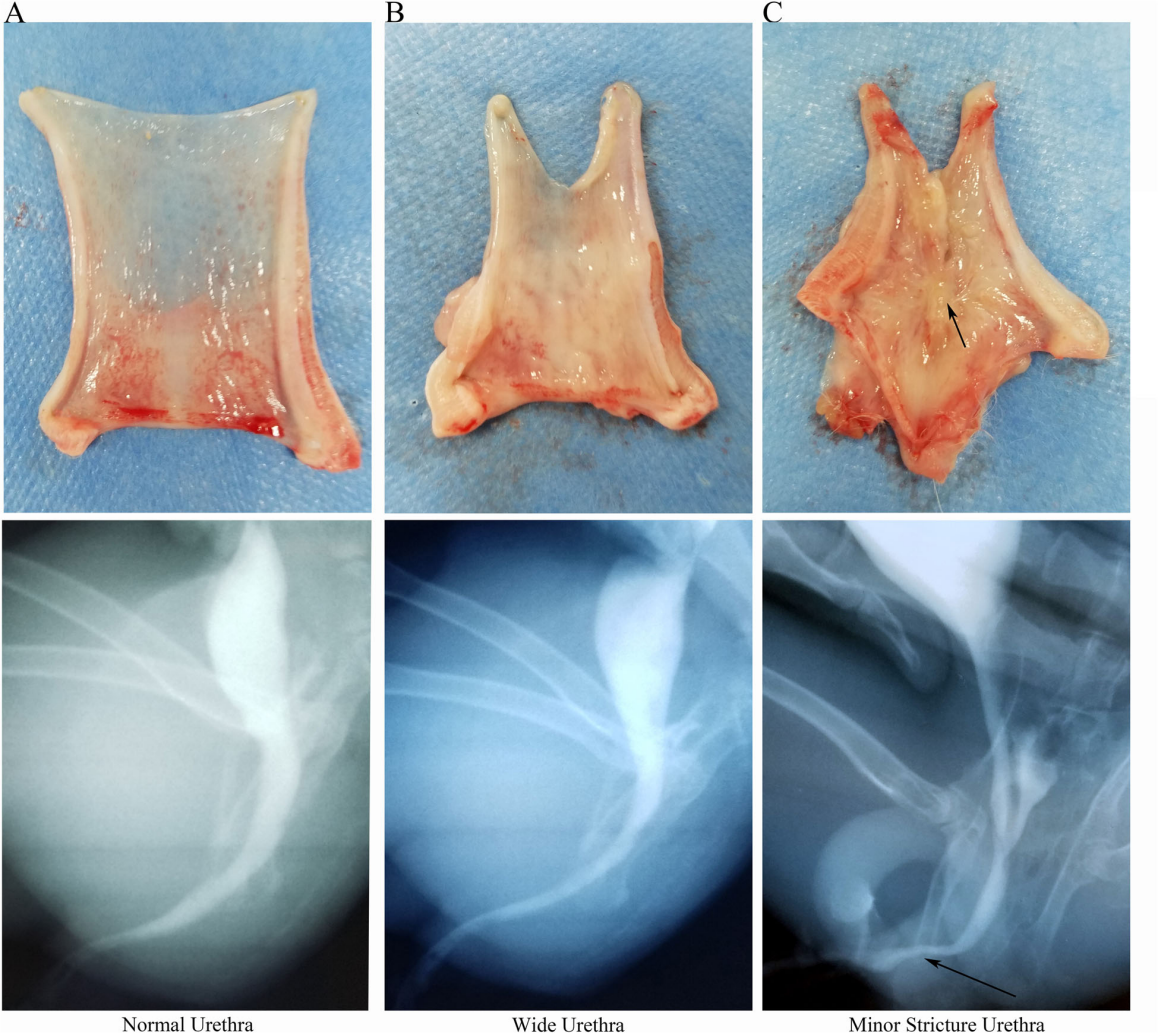
Macroscopy and retrograde urethrograms of retrieved urethras. **a** A normal urethra, **b** a wide urethra in the autologous USC-seeded SIS group, and **c** urethral strictures in the group that received treated SIS alone (*arrow*)

All animals in both groups survived the duration of the study with no abnormal findings. At 3 months, macroscopy and a retrograde urethrogram of the urethra (Fig. 5) showed evidence of strictures. All of the animals treated with 5% PAA-treated SIS alone exhibited strictures, and only one animal in the autologous USC-seeded 5% PAA-treated SIS group exhibited minor strictures.

### Cell labeling and tracking

**Fig. 6:**
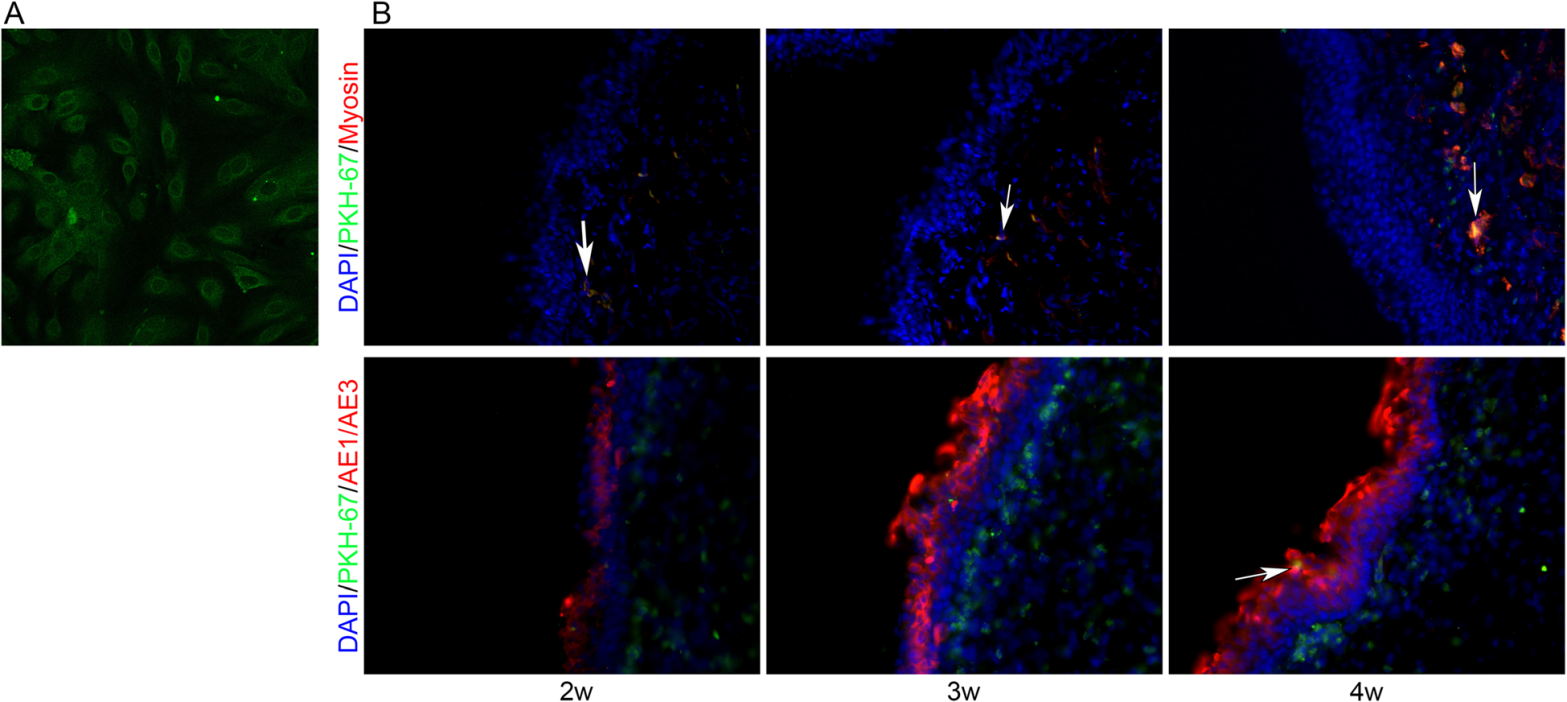
Cell labeling and tracking. USCs were labeled with PKH67 in vitro (**a**) and their differentiation after transplantation was assessed (**b**). Engrafted USCs were positive (*arrows*) for urothelial (AE1/AE3) and smooth muscle (myosin) markers. *DAPI* 4′,6-diamidino-2-phenylindole, *w* weeks

IF staining was used to detect differentiation of the seeded USCs. After labeling, USCs exhibited green fluorescence (Fig. 6a). PKH67-positive USCs were found beneath the luminal surface of the urethra, and some PKH67-positive USCs expressed AE1/AE3 and myosin (Fig. 6b).

### Histopathological evaluation of retrieved urethras

**Fig. 7:**
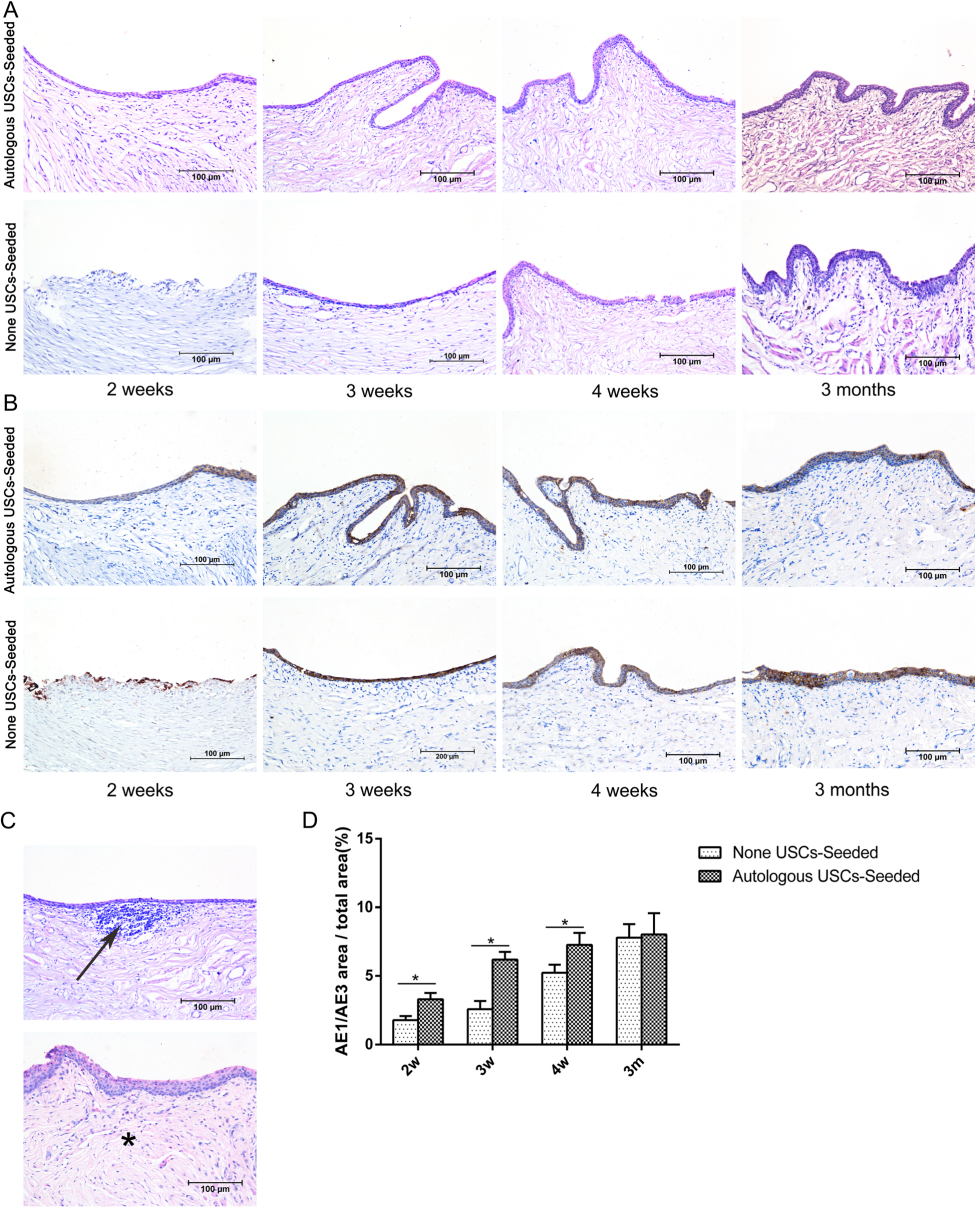
Histopathological evaluation of urothelium regeneration. H&E (**a**) and AE1/AE3 IHC (**b**) staining was used to assess the urothelial regeneration at 2, 3, and 4 weeks and 3 months after transplantation. In the 5% PAA-treated SIS only group, a discontinuous epidermal layer was observed on the luminal surface of the urethra 2 weeks after surgery, and the cellular layer increased over time. At 3 months, the luminal surface had formed an intact and multilayered urothelium. By contrast, complete epidermal cellular layers were formed at 2 weeks in the autologous USC-seeded 5% PAA-treated SIS group, and the urothelium continued to increase over 3 months but then did not change after 3 months. **c** Infiltration of inflammatory cells (*arrow*) and fibrosis (*) were observed in the 5% PAA-treated SIS only group. *Scale bar* = 200 μm. **d** Image analysis was used to calculate the ratio of the AE1/AE3-positive area to the total area at each time point in the two groups. *Significantly lower than in the autologous USC-seeded 5% PAA-treated SIS group at 2, 3, and 4 weeks after transplantation (*P* < 0.05). *m* months, *USC* urine-derived stem cell, *w* weeks

H&E and AE1/AE3 IHC staining (Fig. 7a and b) was used to assess urothelial regeneration. In the group treated with 5% PAA-treated SIS only, a discontinuous epidermal layer was observed on the luminal surface of the urethra 2 weeks after surgery, and the cellular layer continued to increase over time. At 3 months, the luminal surface had formed an intact and multilayered urothelium. However, infiltration of inflammatory cells and fibrocytes (Fig. 7c) was observed, indicating an inflammatory reaction and fibrosis. By contrast, complete epidermal cellular layers were formed at 2 weeks in the group treated with autologous USC-seeded 5% PAA-treated SIS, and the urothelium continued to increase over 3 months but then did not change after 3 months. At 2, 3, and 4 weeks postoperatively, the amount of urothelium was significantly different between the two groups (*P* < 0.05) by histomorphometric analysis (Fig. 7d).

**Fig. 8:**
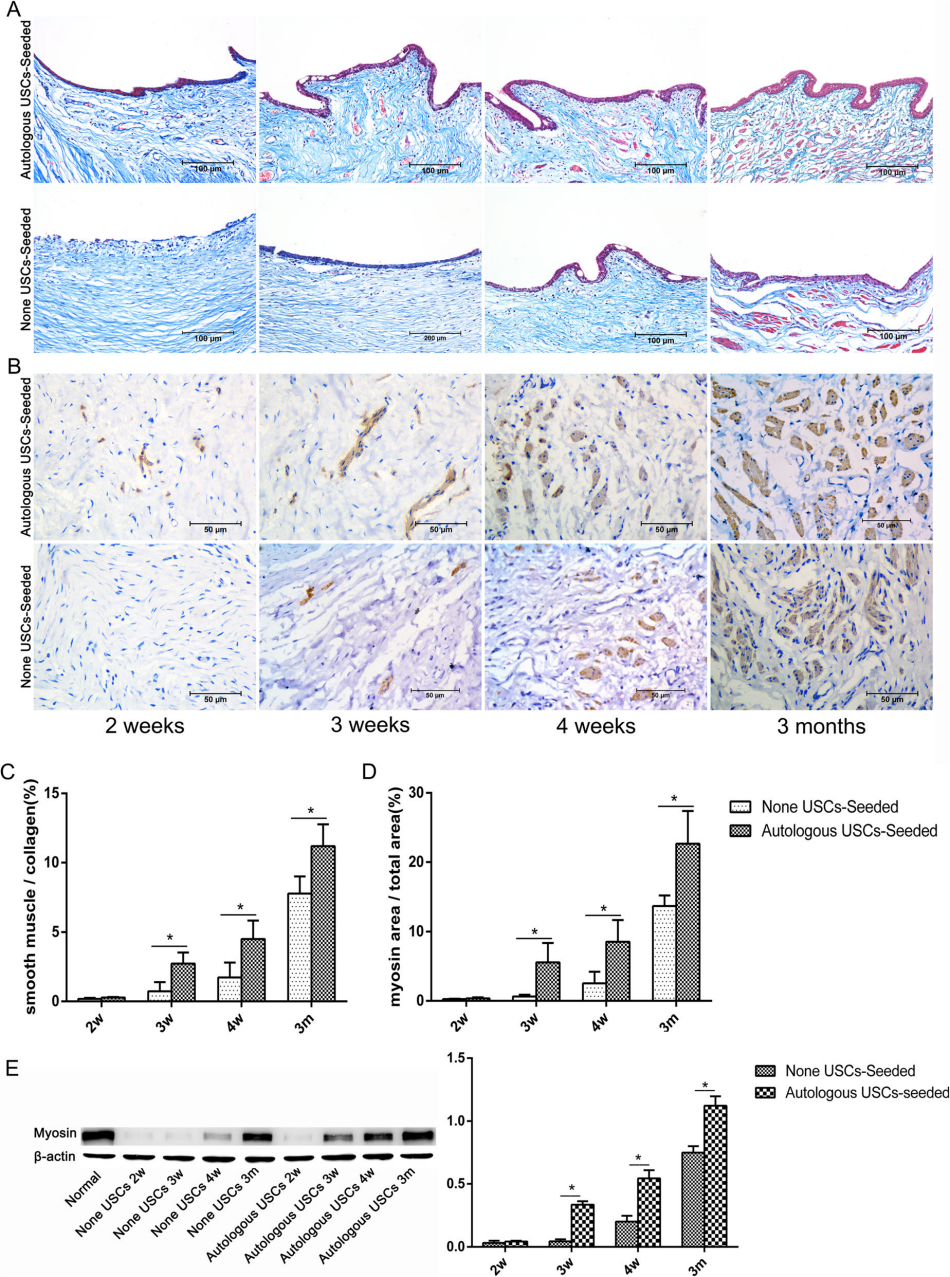
Histopathological evaluation of smooth muscle regeneration. **a**,**b** Smooth muscle regeneration. Representative Masson’s trichrome (**a**) and myosin IHC (**b**) staining in retrieved urethras 2, 3, and 4 weeks and 3 months after transplantation. In the 5% PAA-treated SIS only group, positive expression of myosin beneath the urothelium was observed 3 weeks after the surgery, and the smooth muscle content continued to increase over time. By contrast, expression appeared at 2 weeks in the autologous USC-seeded SIS group. *Scale bar* = 200 μm. **c**,**d** Image analysis. The smooth muscle-to-collagen ratio (**c**) assessed using Masson’s trichrome staining and the smooth muscle-to-total area ratio (**d**) assessed using myosin IHC staining at 3 and 4 weeks and 3 months in the two groups. These parameters were significantly higher in the autologous USC-seeded 5% PAA-treated SIS group than in the 5% PAA-treated SIS only group (*P < 0.05) at 3 and 4 weeks and 3 months. **e** Detection of SMC-specific markers (myosin) by Western blotting. *m* months, *USC* urine-derived stem cell, *w* weeks

Masson’s trichrome and myosin IHC staining (Fig. 8a and b) was used to assess smooth muscle regeneration. In the group treated with 5% PAA-treated SIS only, positive expression of myosin beneath the urothelium was observed 3 weeks after the surgery, and the smooth muscle content continued to increase over time. By contrast, expression was seen at 2 weeks in the group treated with autologous USC-seeded SIS. In addition, the smooth muscle-to-collagen ratio, assessed by Masson trichrome staining, and the smooth muscle-to-total area ratio, assessed by myosin IHC staining, were significantly higher in the autologous USC-seeded 5% PAA-treated group than in the 5% PAA-treated SIS alone group (*P* < 0.05) at 3 and 4 weeks and 3 months (Fig. 8c and d). Furthermore, the smooth muscle became more organized and stained more intensely in the autologous USC-seeded 5% PAA-treated SIS group. Western blot analysis showed that the smooth muscle contents in the two groups were significantly different (Fig. 8e).

**Fig. 9:**
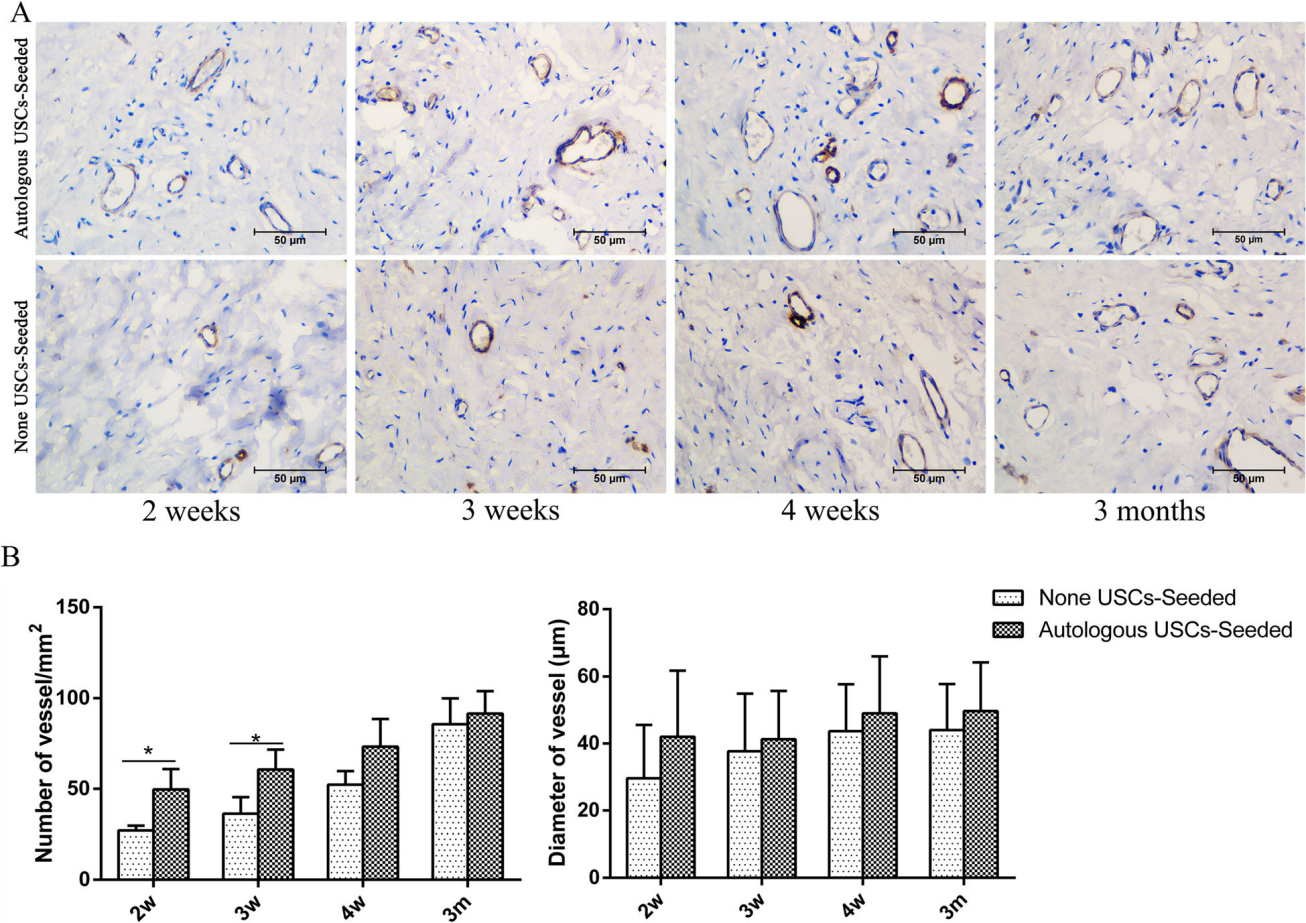
Histopathological evaluation of vascularization. **a** CD31 IHC staining was used to assess vascularization at 2, 3, and 4 weeks and 3 months after transplantation. *Scale bar* = 200 μm. **b** Image analysis was used to quantify the number and diameter of vessels in the two groups at each time point. The number of vessels significantly differed between the two groups at 2 and 3 weeks (*P < 0.05), but the diameter of the vessels was not significantly different. *m* months, *USC* urine-derived stem cell, *w* weeks

Evidence of vascularization assessed using CD31 (Fig. 9a) was also detected in the two groups. The number of vessels significantly differed between the two groups at 2 and 3 weeks (*P* < 0.05), but the diameter of the vessels was not significantly different by histomorphometric analysis (Fig. 9b).

## Discussion

Many donor tissues have been used for urethral reconstruction, including free skin grafts, bladder mucosa, colonic mucosa, buccal mucosa, and lingual mucosa; however, these are not satisfactory and often lead to complications, such as anastomotic strictures and fistulas [20–22]. Furthermore, the use of these tissues requires the harvesting of tissue from a secondary site, and donor site morbidity has been reported [2, 6, 23–26].

Tissue engineering technology provides an alternative approach to build a functional urethra for patients who require urethral reconstruction. Various biodegradable scaffolds have been introduced for urethral reconstruction, including natural collagen materials [8–11] and synthetic polymers such as poly(glycolic acid) [27] and poly(lactic-co-glycolic acid) [28]. However, synthetic biomaterials limit the biologically active molecules that allow tissue regeneration across the graft to proceed, and quickly restrict its application [14]. Natural collagen materials are derived from natural tissues. Their cellular components are removed by a combination of mechanical and chemical manipulation, and extracellular matrix components, such as collagen, fibronectin, laminin, glycosaminoglycans, and growth factors, are retained. They have already been successfully used in urological tissue engineering to treat short urethral defects, both experimentally [12] and clinically [13], with materials such as BSM [8, 9], SIS [10], and collagen type I matrix [11]. However, natural collagen materials also have certain disadvantages that limit their clinical application. After conventional decellularization procedures, SIS often does not possess the highly interconnected macroporous or microporous structures that allow cell infiltration into the matrix, and the material retains some heterogeneous cellular proteins. Despite the high density of collagen and residual heterogeneous cellular proteins, the material can block host cells or seeded cells from infiltrating the collagen matrix and lead to complications. In this study, we combined manual agitation (physical) and Triton X-100 treatment with high concentrations of PAA (chemical) for decellularization of SIS. Similar combinations have been successfully used in previous studies [9, 14, 29]. After treatment with 5% PAA, the porosity and pore size, ranging from 10 to 150 nm on the surface, are higher and larger than SIS without PAA treatment, which allows host cells to infiltrate the SIS scaffold [8] and enhances the transport of nutrients and metabolic waste products [30, 31]. In addition, they are almost completely free of heterogeneous cellular compounds, which could lead to immune reactions, strictures, and fibrosis [29]. Furthermore, the proliferation assay revealed that 5% PAA-treated SIS scaffolds were biocompatible with the USCs. Thus, treatment with 5% PAA contributes to better tissue regeneration.

In addition, the use of acellular matrix is only indicated for short strictures when a healthy and well-vascularized part of the urethral wall exists, with a limit of <1.0 cm for tissue regeneration [32]. Many previous studies [5, 10, 33] have shown that, in urethral reconstruction, cell seeding is necessary to decrease complications when matrices are used. Two types of cells have been introduced for urethral reconstruction. First, adult cells, such as UCs [5, 34, 35], epidermal cells [36], SMCs [37], lingual keratinocytes [37, 38], mesothelial cells [39], and fibroblasts [38], have often been used. However, culturing of these cells has a low success rate, and they also have a limited expansion capability in culture. Second, stem cells, including mesenchymal stem cells [40] and adipose-derived stem cells [27], have been used for tissue engineering. However, these cells have certain disadvantages that limit their clinical application, such as a low differentiation capacity (<5% yield of UCs of endodermal lineage), a short lifespan in vitro (<10 passages for bone marrow stem cells), and the need for invasive collection procedures [16]. In this experiment, we were able to harvest autologous USCs and expand them to sufficient numbers for scaffold seeding using a simple, safe, low-cost, and non-invasive procedure. USCs possess both self-renewal and multilineage differentiation capabilities; they can differentiate into osteocytes, chondrocytes, adipocytes, skeletal myocytes, interstitial cells, and endothelial cells [15, 18]. We demonstrated that USCs were able to differentiate into cells of the smooth muscle lineage (mesodermal origin) and urothelial lineage (endodermal origin) in vitro; therefore, USCs may assume the role of urethral SMCs and UCs. Because the cells were to be grafted into recipients, immunological rejection should be considered. However, autologous cells have no issues relevant to immunological rejection. In addition, as a type of stem cell, USCs also can regulate the immune response by inhibiting inflammatory and immunomodulatory factors.

Previous studies [14, 41] have shown that a 3D porous biological collagen matrix provides favorable conditions for USC expansion and differentiation, and that USC-seeded 3D porous biological collagen matrix can be used to construct a tissue-engineered urethra. It is not clear whether USC-seeded 3D porous biological collagen matrices are an effective therapy in vitro. Data from previous studies [9, 14, 41] were used as a foundation to extend the use of autologous USCs seeded on a 3D porous SIS to construct a tissue-engineered urethra. In this study, we investigated the feasibility and availability of an autologous USC-seeded 3D-porous SIS scaffold to reconstruct the urethra.

In this study, H&E and DAPI staining revealed that autologous USCs were successfully seeded into 3D porous SIS scaffolds on both sides, and the cells attached well and formed a multilayer of cells on the surface of the SIS. Additionally, H&E and AE1/AE3 IHC staining showed integrated epidermal cell layers in the autologous USC-seeded 3D porous SIS scaffold group 2 weeks after the operation. By comparison, stratified epidermal cellular layers were formed at 3 weeks. Despite the infiltration of the host epithelial cells, there was no significant difference in urothelial regeneration between the two groups at 3 months. However, the early integrated epidermal cellular layers attached to the luminal surface of the urethra may provide a permeability barrier that can prevent urine leakage from the underlying tissues, which is one of the major factors implicated in the inflammatory infiltration and fibrous tissue deposition that lead to stricture formation. Importantly, Masson’s trichrome, myosin IHC staining, and functional analysis showed that the animals that received the autologous USC-seeded 3D porous SIS scaffold showed improvement over those that received the 3D porous SIS scaffold alone. SMCs were seen in the autologous USC-seeded 3D porous SIS scaffold group 2 weeks postoperatively. These continued to develop and approximate normal tissues throughout the study with no abnormal findings. By comparison, SMCs began to appear at 3 weeks postoperatively. The smooth muscle-to-collagen ratio and the content of smooth muscle were significantly higher in the autologous USC-seeded 3D porous SIS group than in the 3D porous SIS alone group (*P* < 0.05) at 3 and 4 weeks and 3 months. Additionally, all of the animals in the 3D porous SIS alone group exhibited strictures, and only one animal in the autologous USC-seeded 3D porous SIS group exhibited minor strictures. The number of vessels was significantly higher in the autologous USC-seeded 3D porous SIS group than in the 3D porous SIS alone group at early time points, which allowed host cells to infiltrate the scaffold and enhanced the transport of nutrients and metabolic waste products, thereby promoting urethral regeneration. We also demonstrated that the native urethra environment in the animal appeared to provide enough stimuli to cause the USCs to differentiate into SMCs and UCs and to assume the role of urethra SMCs and UCs. In addition, USCs secrete a variety of growth factors, such as vascular endothelial growth factor (VEGF), PDGF, PLGF, basic fibroblast growth factor, epidermal growth factor, and TGF-β [42]. VEGF expression enhances muscle regeneration in vivo [43]. This might lead to the recruitment of host cells, a direct effect of angiogenesis, and contribute to the smooth muscle and urothelial layers that repopulate the urethra. These findings indicate that autologous USCs can promote urothelium and smooth muscle regeneration and vessel infiltration and decrease the indwelling time, thus reducing complications, such as infection, scarring, and strictures.

In this study, however, inflammatory cell infiltration and fibrosis were found in the SIS alone group. In addition, all animals in the SIS alone group exhibited strictures, and one animal in the autologous USC-seeded group exhibited minor strictures. Due to the immunomodulatory and immunosuppressive properties of USCs, it is believed that they can secrete immunomodulatory factors after transplantation, including interleukins (ILs; such as IL-2 and IL-12), cytokines (such as interferon and granulocyte colony-stimulating factor), and chemokines (such as macrophage inflammatory protein-1α) [42], and inhibit peripheral blood mononuclear cell (T and B cell) proliferation [44]. Moreover, in control animals, in which USCs were not seeded on the scaffolds, immunoreaction was one of the major factors implicated in the inflammatory cell infiltration that led to stricture formation. Another contributing factor to the success of urethral reconstruction could be the permeability barrier formed by the USCs. For these reasons, histological analysis and the retrograde urethrogram did not show an immune reaction, an inflammatory response, or stricture formation within the autologous USC-seeded 3D porous SIS scaffold group.

Although this study provides evidence that autologous USC-seeded 3D porous SIS scaffolds can be used to construct a tissue-engineered urethra, it has some limitations. First, we did not analyze the bioactive factors, such as growth factors and cytokines, that are secreted by USCs and which are believed to enhance tissue regeneration. Second, in the present study, the urethral defect models were established in normal animals, which cannot fully simulate the exact clinical situation of urethral strictures, which is characterized by a fibrotic urethral bed. Finally, the number of experimental animals that were evaluated per time point is small. Further investigations are necessary to translate this technology into the clinic.

## Conclusions

USCs that can be easily isolated from voided urine can be extensively expanded to high numbers in vitro, and efficiently give rise to UCs and SMCs in vitro and vivo. They can be utilized as a promising cell source for tissue-engineered urethras. In addition, 3D porous SIS as an optimized biomaterial has potential as a cell-based scaffold for tissue-engineered urethra. Furthermore, an autologous USC-seeded 3D porous SIS scaffold could promote urethral regeneration.

## Abbreviations

BSM: Bladder submucosa
DAPI: 4′,6-Diamidino-2-phenylindole
EFM: Embryonic fibroblast medium
H&E: Hematoxylin and eosin
IF: Immunofluorescence
IHC: Immunohistochemistry
IL: Interleukin
PAA: Peracetic acid
PBS: Phosphate-buffered saline
PDGF: Platelet-derived growth factor
SIS: Small intestinal submucosa
SMC: Smooth muscle cell
TGF: Transforming growth factor
UC: Urothelial cell
USC: Urine-derived stem cell
VEGF: Vascular endothelial growth factor
